# Trauma admissions to the Intensive care unit at a reference hospital in Northwestern Tanzania

**DOI:** 10.1186/1757-7241-19-61

**Published:** 2011-10-24

**Authors:** Phillipo L Chalya, Japhet M Gilyoma, Ramesh M Dass, Mabula D Mchembe, Michael Matasha, Joseph B Mabula, Nkinda Mbelenge, William Mahalu

**Affiliations:** 1 Department of Surgery, Weill-Bugando University College of Health Sciences, Mwanza, Tanzania; 2 Department of Orthopaedics, Weill-Bugando University College of Health Sciences, Mwanza, Tanzania; 3 Department of Surgery, Muhimbili University of Health and Allied Sciences, Dar Es Salaam, Tanzania; 4 Department of Anaesthesia, Weill-Bugando University College of Health Sciences, Mwanza, Tanzania; 5 Cardiothoracic unit, Weill-Bugando University College of Health Sciences, Mwanza, Tanzania

**Keywords:** Intensive care unit, trauma admissions, prevalence, injury characteristics, outcome, Tanzania

## Abstract

**Background:**

Major trauma has been reported to be a major cause of hospitalization and intensive care utilization worldwide and consumes a significant amount of the health care budget. The aim of this study was to describe the characteristics and treatment outcome of major trauma patients admitted into our ICU and to identify predictors of outcome.

**Methods:**

Between January 2008 and December 2010, a descriptive prospective study of all trauma admissions to a multidisciplinary intensive care unit (ICU) of Bugando Medical Centre in Northwestern Tanzania was conducted.

**Results:**

A total of 312 cases of major trauma were admitted in the ICU, representing 37.1% of the total ICU admissions. Males outnumbered females by a ratio of 5.5:1. Their median age was 27 years. Trauma admissions were almost exclusively emergencies (95.2%) and came mainly from the Accident and Emergency (60.6%) and Operating room (23.4%). Road traffic crash (RTC) was the most common cause of injuries affecting 70.8% of patients. Two hundred fourteen patients (68.6%) required surgical intervention. The overall ICU length of stay (LOS) for all trauma patients ranged from 1 to 59 days (median = 8 days). The median ICU length of hospital stay (LOS) for survivors and non-survivors were 8 and 5 days respectively. (P = 0.002). Mortality rate was 32.7%. Mortality rate of trauma patients was significantly higher than that of all ICU admissions (32.7% vs. 18.8%, P = 0.0012). According to multivariate logistic regression analysis, multiple injuries, severe head injuries and burns were responsible for a longer mean ICU stay (P < 0.001) whereas admission Glasgow Coma Score < 9, systolic blood pressure < 90 mmHg, injury severity core >16, prolonged duration of loss of consciousness, delayed ICU admission (0.028), the need for ventilatory support and finding of space occupying lesion on computed tomography scan significantly influenced mortality (P < 0.001).

**Conclusion:**

Trauma resulting from road traffic crashes is a leading cause of intensive care utilization in our hospital. Urgent preventive measures targeting at reducing the occurrence of RTCs is necessary to reduce ICU trauma admissions in this region. Improved pre- and in-hospital care of trauma victims will improve the outcome of trauma patients admitted to our ICU.

## Background

Major or severe trauma constitutes a global public health problem and poses diagnostic and therapeutic challenges to trauma, orthopaedic and general surgeons practicing in developing countries [1]. Major trauma contributes significantly to high morbidity, mortality and long-term disabilities worldwide [1,2]. In developing countries including Tanzania, injuries in general are increasing due to increase in urbanization, motorization, civil violence, wars and criminal activities [3]. In these countries, major trauma remains a major cause of hospitalization and intensive care utilization and consumes a significant amount of the health care budget [4]. In Bugando Medical Centre, major trauma is the single most common reason for admission to the Intensive Care Unit (ICU) and is it associated with substantial emotional, physical and financial burden on community and hospital resources.

Major trauma is often life threatening and usually presents as an emergency, requiring either immediate surgical intervention or intensive care or both [4,5]. The Intensive Care Unit is a specialized area where facilities for the critically ill or severely injured patients are concentrated and where the level of care and supervision is considerably more sophisticated than in the ordinary ward [4-6]. Worldwide, intensive care unit requires a vast use of up to date resources such as advanced monitors, organ support equipments and highly skilled staff. This however, often taxes the most buoyant health systems even of the developed nations [4,6]. In most developing nations where there are several financial constraints resulting from poor funding of the health care generally and the ICU specifically, there is often a limit to the availability and specialization of this form of care [6]. Besides allocation of resources, intensive care also demands a tremendous amount of time and effort on behalf of the medical and nursing staff to treat and improve survival of the critically ill patients. It therefore follows that the role of the ICU must be justified wherever it exists [5,6]. The allocation of ICU facilities when financial resources are limited is determined by cost/benefit and patient outcome [6,7]. Admission of patients with poor prognosis and/or prolonged use of the ICU facility results in other patients with a better prognosis being denied care; many of these die as a result [7].

Despite continued advances in intensive care unit technology and the availability of sophisticated interventions for the treatment of critically ill or severely injured patients, major trauma patients continue to die in the ICU [6,7]. Identification of factors responsible for this state of affairs is of paramount in order to improve the outcome these patients. Understanding the magnitude of the problem and characterizing the patterns of injury in these patients is important in planning programmes targeted at preventing their occurrence and subsequently reduce ICU admissions. There is paucity of published data on ICU trauma admissions in our environment despite large number of trauma admissions to our ICU. This study was conducted to describe the characteristics and treatment outcome of major trauma patients admitted into our ICU and to identify predictors of outcome.

## Methods

### Study design and setting

Between January 2008 and December 2010, a descriptive prospective study was conducted in the ICU of Bugando Medical Centre (BMC). BMC is found in Mwanza city in the Northwestern Tanzania along the shore of Lake Victoria. It is one of the four tertiary referral hospitals in the country and serves as a teaching hospital for Weill- Bugando University College of Health Sciences (WBUCHS) and other paramedics. It has a bed capacity of 1000 and provides services to approximately 13 million people from six regions namely Mwanza, Kagera, Mara, Shinyanga, Kigoma and Tabora. The hospital has a 12-bed adult and 10-bed paediatric multi-disciplinary Intensive Care Unit (ICU) which is headed by a consultant anesthesiologist and run by trained ICU nurses. The ICU provides services to all patients (trauma and non-trauma, medical and surgical) requiring advanced airway support, mechanical ventilation, hemodynamic support, and electronic monitoring which are usually not available in the open wards in our hospital. The majority of trauma patients admitted in the ICU come from the Accident and Emergency (A & E) department, operating theatre, wards and others come from other peripheral hospitals.

### Study subjects

The study subjects included all patients with traumatic injuries severe enough to warrant ICU admission and who consented for the study. Patients who were readmitted to the ICU during the same hospital stay were excluded from the study. A total of 334 patients were recruited for the study and screened for inclusion criteria. Of these, 314 patients met the inclusion criteria and the remaining 20 patients did not. Patients who met the inclusion criteria were requested to consent for the study before being enrolled in the study. Two patients refused to consent for the study. Thus, 312 patients were enrolled in the study.

All study patients were initially resuscitated according to ATLS and treated according to ICU protocols. The severity of injury was assessed by the injury severity score (ISS) and the Glasgow coma score (GCS). An initial systolic blood pressure (SBP) on each patient was also recorded on admission. Data were collected used a questionnaire. Data administered in the questionnaire included details of demographic profile, causes of injury, injury characteristics, injury severity using Glasgow Coma scale (GCS) and injury severity score (ISS), treatment offered, complications, ICU length of stay (LOS), mortality and patient disposal. ICU length of stay. Patients were followed up till death or discharge from the ICU.

### Statistical data analysis

Data collected was analyzed using SPSS software version 15.0. Data was summarized in form of proportions and frequent tables for categorical variables. Continuous variables were summarized using means, median, mode and standard deviation. χ2-test was used to test for significance of associations between the predictor and outcome variables in the categorical variables. Student t-test was used to test for significance of associations between the predictor and outcome variables in the continuous variables.. Significance was defined as a p-value of < 0.05. Multivariate logistic regression analysis was used to determine predictor variables that are associated with outcome.

#### Variable definitions

Road traffic crash was defined as accident which took place on the road between two or more objects one of which must be any kind of a moving vehicle.

Patients were divided into two groups according to the timing of ICU admission on arrival. Patients who were admitted to the ICU immediately on arrival were classified as "immediate admission group" and patients who had to wait for ICU admission due to bed unavailability or any other reason were classified as "delayed admission group". The nature of injury was classified as "intentional injuries" defined as injuries that occur as a result of interpersonal or self-inflicted violence and "unintentional injuries" defined as injuries that occur accidentally. ICU length of stay was arbitrary dichotomized into two groups' i. e. ≤ 14 days and > 14 days respectively. An ICU length of stay of > 14 days was considered as prolonged hospital stay.

### Ethical consideration

Ethical approval to conduct the study was obtained from the WBUCHS/BMC joint institutional ethic review committee before the commencement of the study. Patients who met the inclusion criteria or their relative were requested to sign a written informed consent before being enrolled into the study.

## Results

There were a total of 312 trauma admissions to the ICU during the period under study, representing 37.1% (312/841) of the total ICU admissions. There were 264 (84.6%) males and females were 48 (15.4%) with a males to female ratio of 5.5:1 compared with 1.2:1 for the total ICU admissions. Their ages ranged from 4 years to 71 years (median 27 years). Trauma admissions were almost exclusively emergencies (95.2%) and came mainly from the Accident and Emergency (60.6%) and Operating Room/Post Anaesthesia Care Unit (23.4%). (Table 1)

**Table 1:** Demographic and clinical characteristics of the ICU trauma admissions

Study variable	Trauma admission	Total admission
**Number (N/%)**	312 (37.1)	841 (100)
**Sex (Male: Female ratio)**	5.5: 1	1.2:1
**Median age (in years)**	27	30
**Modal age group (years)**	21-30	31-40
**Source of admission (N/%)**		
° A & E department	189 (60.6)	301 (35.8)
° OR/PACU	73 (23.4)	385 (45.8)
° Wards	33 (10.3)	132 (15.7)
° Other hospital s	17 (5.7)	22 (2.6)
° Unspecified	-	1 (0.1)
**Timing of admission**		
° Immediately	111 (35.6)	365 (43.4)
° Delayed	201 (64.4)	476 (56.6)
**Type of admission (N/%)**		
° Emergency	297 (95.2)	573 (68.1)
° Elective	15 (4.8)	268 (31.9)
**Length of hospital stay (N/%)**		
° < 3 days	110 (35.1)	370 (44.0)
° ≥ 3 days	202 (64.9)	471 (56.0)
**Mortality rate (%)**	102 (32.7)	158 (18.8)

Keys: A & E = Accident and Emergency; OR = Operating Room; PACU = Post Anaesthesia Care Unit

The majority of the injuries were unintentional in 232 (74.4%) patients. Intentional injuries were recorded in sixty (19.2%) of cases and the remaining twenty (6.4%) patients were cases of indeterminate intent. Road traffic crush was the most common cause of injuries affecting 70.8% of patients (Table 2).

**Table 2:** Causes of injuries among ICU trauma victims

Cause of injury	Number of patients	Percentage
Road traffic crush	221	70.8
Assaults	36	11.5
Falls	12	3.8
Burns	9	2.9
Other causes	14	4.5
Unknown	20	6.4
**Total**	**312**	**100**

One hundred and fifty-two (68.8%) of RTCs were related to motorcycle injuries affecting motorcyclists (93, 61.2%), passengers (40, 26.3%) and pedestrian (19, 12.5%).

The head/neck and musculoskeletal (extremities) regions were commonly affected accounting for 95.5% and 34.6% of cases respectively (Table 3). Isolated injuries occurred in 208 (66.7%) patients while 104 (33.3%) patients had multiple injuries. Soft tissue injuries (i.e. bruises, laceration, abrasion and contusions) and fractures (long bones, spines, pelvis, ribs, and skull) were the most common type of injuries accounting for 97.8% and 32.4% respectively **(**Table 4).

**Table 3:** Site of injury among ICU trauma victims

Site of injury	Frequency	Percentage
Head/neck	298	95.5
Musculoskeletal (extremities)	108	34.6
Chest	82	26.3
Abdomen	62	19.9
Pelvis	14	4.5
Spines	8	2.6
Genitalia	4	1.3

**Table 4:** Type of injuries among ICU trauma victims

Type of injury	Frequency	Percentage
Soft tissue injuries (wounds)	305	97.8
Fractures	101	32.4
Craniocerebral injury	67	21.5
Visceral injury	41	13.1
Burns	9	2.9
Other injuries	12	3.8

The ISS ranged from 16-56 with the mean of 19.74 ± 9.81. The median was 17.00. The mean GCS for patients with head injuries was 9.65 ± 12.43 (range 3-15). The median GCS was 9. The majority of patients (172, 55.1%) had admission SBP < 90 mmHg.

Of the 312 patients admitted to the ICU, 169 (54.2%) patients were intubated and ventilated for a median of 7 days (range 1-32 days). Two hundred fourteen patients (68.6%) required surgical intervention. Wound debridement, treatment of fractures and craniotomies were the commonest surgical procedures performed in 95.3%, 43.5% and 14.5% of patients respectively (Table 5).

**Table 5:** Type of surgical procedure performed (N = 214)

Surgical procedure	Frequency	Percentages
Wound debridement	204	95.3
Treatment of fractures	93	43.5
Craniotomy (including elevation of skull fracture)	31	14.5
Underwater seal drainage	23	10.7
Exploratory laparotomy	17	7.9
Tracheostomy	12	5.6
Eye surgery	9	4.2
Thoracotomy	2	0.9

The overall ICU length of stay (LOS) for all trauma patients ranged from 1 to 59 days (median = 8 days). The median ICU length of hospital stay (LOS) for survivors and non-survivors were 8 and 5 days respectively. These differences were statistically significant (P = 0.002).

Of the non-survivors, ten (9.8%) died within 24 hours of ICU admission while twenty-four (23.5%) died within 72 hours and six-eighty (66.7%) died by the seventh ICU day. Of the survivors, thirty-two (15.2%) were discharged to the wards within 72 hours, fifty-four (25.7%) on the seventh day and one hundred and twenty-four (59.0%) were discharged by the fourteenth day of ICU admission.

Analysis of outcome showed that 201(64.4%) patients were transferred to the ward and 4 (1.3%) patients were discharged home direct from the ICU. 3 patients (1.0%) were referred to another tertiary institution and 1 (0.3%) patient each absconded and left against medical advice respectively. A total of 102 patients died giving a mortality rate of 32.7%. Mortality rate of trauma patients was significantly higher than that of all ICU admissions (32.7% vs. 18.8%, P = 0.0012). According to multivariate logistic regression analysis, multiple injuries (O.R.= 2.34, 95% C.I.( 2.11-4.78), P = 0.012), severe head injuries [GCS = 3-8] (O.R.= 0.54, 95% C.I.(0.13-0.69), P = 0.036) and burns (O.R.= 4.92, 95%C.I. (2.43-8.15), P = 0.017) were responsible for a longer (> 14 days) ICU stay. Table 6 shows predictors of mortality according to univariate and multivariate analysis

**Table 6:** Predictors of mortality according to univariate and multivariate analysis

Independent (predictor)variable	Number of patients (N/%)	Univariate analysis		Multivariate analysis	
		
		OR. (95%C.I)	p-value	OR. (95%C.I)	p-value
**Age**					
≤40	202(64.7)	1		1	
> 40	110(35.3)	1.41 (0.45-1.82)	0.098	2.5 (0.56-6.67)	0.765
**Sex**					
Male	264(84.6)	1		1	
Female	48 (15.4)	4.52 (0.95-5.83)	0.678	3.12(0.97-5.78)	
**Type of admission**					
Emergency	297(95.2)	1		1	
Elective	15 (4.8)	2.71 (1.2-3.7)	0.024	3.83 (0.93-5.98)	0.934
**Timing of admission**					
Immediately	111 (35.6)	1		1	
Delayed	201 (64.4)	2.93 (2.22-8.45)	0.003	3.43(2.26-7.91)	**0.028**
**Duration of LOC (N = 214)**					
≤ 2 hours	72(33.6)	1		1	
> hours	146(68.2)	2.23 (1.89-7.98)	0.000	0.34(0.12-0.78)	**0.004**
**ISS**					
≤ 15	2(0.6)	1		1	
>15	310 (99.4)	6.83(3.56-8.91)	0.012	7.21 (2.65-10.96)	**0.000**
**GCS**					
<9	192(61.5)	1			
≥9	120(38.5)	2.64( 1.32-6.76)	0.034	4.72 (3.16-9.23)	**0.003**
**Admission SBP(mmHg)**					
< 90	172(55.1)	1		1	
≥90	140(44.9)	1.76(0.56-4.35)	0.042	8.34 (4.61-9.98)	**0.016**
**Need for ventilatory support**					
Yes	169(54.2)	1		1	
No	143 (45.8)	2.45(1.24-5.89)	0.038	0.65 (0.28-0.86)	**0.018**
**CT scan brain findings of SOL (N = 122)**					
Yes	98(80.3)	1		1	
No	24(19.7)	0.77 (0.23-0.98)	0.000	3.53(2.23-6.97)	**0.015**

Abbreviations: O.R.= Odds ratio; C.I.= confidence interval; LOC = loss of consciousness; ISS = injury severity score; GCS = Glasgow coma scale; SBP = systolic blood pressure; SOL = space occupying space

## Discussion

### Number of ICU trauma admission

In this review, major trauma was the most common indication for admissions into our ICU (37.1%), and represented 95.2% of emergency ICU admissions. This is higher than that reported in Jamaica and Nigeria by Mitchell *et al *[7] and Amanor-Boadu *et al *[8] respectively. ICU admission has been reported to be the most important factor in determining the ultimate outcome of critically ill and major trauma patients and to be successful requires adequate logistic and financial support, supporting disciplines (e.g. laboratories, radiology and surgery) and basic infrastructure such as good roads, regular electricity, water supply, availability of drugs by the patient's bedside and regular oxygen and compressed air supply [9]. Many of these are not regularly available in many low resource settings. It has been suggested that the inadequate medical and technical equipment of most ICUs in low resource economies substantially contribute to the high mortality rate of critically ill patients in such countries [10]. Facilities in our ICU are limited and obviously insufficient to cope with the number of patients being admitted.

### Demographic profile

In agreement with previous studies [5-8,11], trauma patients admitted to our ICU were mostly young males, and had a better previous health status than most other ICU patients. However, the mortality was higher among them compared to the non-trauma patients. This group represents the economically active age and portrays an economic lost both to the family and the nation and the reason for their high incidence of traumatic injuries reflects their high activity levels and participation in high-risk activities. Male predominance in the present study is due to their increased participation in high-risk activities. The fact that the economically productive age-group were mostly involved calls for an urgent public policy response.

### Timing of ICU admission

In keeping with other studies [12,13], our study has demonstrated an association between delay to ICU admission and higher mortality rate reflecting worsening of organ dysfunction during this period. Despite the care provided by ward healthcare workers while patients were waiting for ICU bed availability, these healthcare providers were not trained in critical care and were not as experienced in caring for ICU patients. Furthermore, hospital wards are neither designed nor staffed to provide extended longitudinal care for the critically ill patient. These patients have better outcomes when treated in ICUs with close and continuous involvement by critical care physicians [14,15] and other data also show improved outcome when nurse-to-patient ratios in the ICUs are properly maintained [16]. Caring for critically ill patients outside the ICU may also imply an increased burden and high stress level experienced by hospital wards staff.

### Etiological pattern

Road traffic crushes have been reported to be the commonest cause of ICU trauma admissions in most studies as supported by the present study [7,8,11]. This may be attributed to recklessness and negligence of the driver, poor maintenance of vehicles, driving under the influence of alcohol or drugs and complete disregard of traffic laws. In the present study, motorcycle injuries accounted for more than fifty percent of road traffic injuries and the motorcyclist constituted the majority of motorcycle injury victims. Findings from this study calls for urgent interventions targeting at reducing the occurrence of RTCs and subsequently reduce the incidence of ICU admissions in this region.

### Type of injuries

The majority of our patients sustained head and musculoskeletal injuries, which is in keeping with previous studies [6-8,17-19]. Severe road traffic crashes are usually associated with significant head, musculoskeletal and multiple injuries, which explain why they are the leading cause of our ICU admissions, unlike in the wards.

### Trauma scores

The severity of injury on admission in the present study was assessed by the ISS and the GCS. Most validated ICU scoring system such as admission APACHE (Acute physiology age and chronic health evaluation) II Score were not used in this study because the majority of patients were transferred to the unit after initial stabilization from the Accident and Emergency unit. Also APACHE II score use parameters that require arterial blood gas analysis and other variables that we were unable to measure.

### Length of ICU stay

In this study, survivors had a statistically significant longer LOS than non-survivors which is in agreement with other studies in developing countries [7,8,20], but contrary to studies in developed countries which reported non-survivors staying longer and consuming more resources than survivors [10,21]. This difference in survival is probably due to a combination of factors including severity of injuries, poor pre-hospital care, lack of emergency medical services, and lack of appropriate diagnostic and therapeutic facilities including drugs for the care of these patients in the hospital and the ICU. These factors have been adequately addressed in developed nations.

### Mortality

Our mortality rate (32.7%) in the present study was higher than the mortality rate of 26.4% reported by Mitchel *et al *[7] in Jamaica but lower compared to mortality rate of 53.2% reported by Adenekan *et al *[20] in Nigeria. Higher mortality rate in our study may be attributed to severity of injuries, lack of advanced pre-hospital care in our setting, ineffective ambulance system for transportation of patients to hospitals, and lack of appropriate diagnostic and therapeutic facilities including drugs for the care of these patients in the hospital and the ICU. This observation calls for improved pre- and in-hospital care of trauma victims so as to improve the outcome of trauma patients admitted to our ICU.

### Study limitations

Despite limitations of the study such as limited ICU space, unavailability of diagnostic tools (e.g. Computed tomography scan) and short-term study limited to ICU stay, this study demonstrated the impact of trauma admissions to our ICU.

## Conclusion

Major trauma resulting from road traffic crashes is a leading cause of intensive care utilization in our hospital. Urgent preventive measures targeting at reducing the occurrence of RTCs is necessary to reduce the incidence of major trauma in this region and subsequently reduce ICU trauma admissions. These preventive measures include increased public education, enforcement of road safety rules, improvement in socioeconomic situation and employment opportunities in our country. Improved pre- and in-hospital care of trauma victims will improve the outcome of major trauma patients admitted to our ICU.

## Competing interests

The authors declare that they have no competing interests.

## Authors' contributions

PLC contributed in study design, literature search, data analysis, manuscript writing & editing. JMG and RMD participated in study design, data analysis, manuscript writing & editing. MDM participated in data analysis, manuscript writing & editing. MM and JBM participated in data analysis and manuscript writing. WM supervised the study and contributed in data analysis, manuscript writing & editing. All the authors read and approved the final manuscript.
